# Understanding implementation of the ‘high volume, low complexity’ surgical hubs programme in England: a qualitative study

**DOI:** 10.1136/bmjopen-2026-119396

**Published:** 2026-07-17

**Authors:** Pete Lampard, Arabella Scantlebury, Peter Sivey, Joy Adamson

**Affiliations:** 1 York Trials Unit, Department of Health Sciences, University of York, York, UK; 2 Centre for Evidence and Implementation Science, University of Birmingham, Birmingham, UK; 3 Centre for Health Economics, University of York, York, UK

**Keywords:** surgical hubs, implementation, elective surgery, innovation, service reconfiguration

## Abstract

**Abstract:**

**Objective:**

To explore the implementation of England’s high volume, low complexity (HVLC) surgical hubs programme from the perspective of senior leaders involved in its development and rollout.

**Design:**

A qualitative design using semistructured interviews with service leaders.

**Setting:**

Following the postponement of elective surgical care in England after the outbreak of COVID-19, NHS England and the ‘Getting it Right First Time’ (GIRFT) programme developed an elective recovery plan for the NHS, with surgical hubs as the chief innovation for building elective surgical capacity.

**Participants:**

12 service leaders, directly involved in the development and/or implementation of the HVLC surgical hubs programme.

**Results:**

From the service leader interviews, four interrelated themes explained the rapid diffusion of surgical hubs: a ‘window of opportunity’ created by COVID-19, which accelerated adoption by reframing elective backlog as an acute crisis and enabling targeted investment; leadership by respected clinicians and the use of peer-generated, data-driven protocols which built trust and engagement; an iterative, locally adaptive approach to implementation, framing oversight as supportive rather than punitive; and peer-to-peer accreditation, allowing for the showcasing of best practice and fostering a growing network for continued shared learning.

**Conclusion:**

The implementation of high-volume, low-complexity surgical hubs in England demonstrates how large-scale service reconfiguration can be accelerated under crisis conditions but sustained through clinical credibility, trusted data and improvement-focused oversight. Our findings highlight the importance of hybrid governance models that combine national direction with local adaptability, supported by peer networks and accreditation mechanisms. These features align with wider evidence on health system change, underscoring the need for organisational readiness, relational work, and supportive infrastructures to ensure long-term impact beyond initial investment and leadership momentum. Future research should explore how these hubs function in practice, and whether the views of surgical hub staff align with those given by service leaders.

**STRENGTHS AND LIMITATIONS**

- A rich source of data: this study used interviews with those specifically involved in the development and implementation of the high volume, low complexity (HVLC) surgical hubs policy and programme, many of which involved those in high-level positions within NHS England.
- A combination of purposeful and snowball sampling provided a diversity of perspectives on surgical hubs, though all were broadly positive; future work should explore those not involved in the development and implementation of the hubs, where dissenting voices are more likely to be found.
- The development and implementation of the HVLC surgical hubs policy follows closely after the early waves of COVID-19 and special care must be taken when translating findings from this period to others.

## Introduction

 Health systems worldwide have faced mounting pressures on elective surgical capacity for over a decade, with waiting lists lengthening further in the wake of the COVID-19 pandemic.^1 2^ In England, this compelled NHS England to produce an elective recovery plan, advocating service reconfiguration through the adoption of high volume, low complexity (HVLC) surgical hubs. The hubs—dedicated, ring-fenced sites for common, lower complexity procedures (eg, cataracts, hip replacements)—were introduced as the primary strategy to increase elective surgical throughput and reduce waiting times.^3^

Though the practice of ring-fencing elective from emergency surgical care is not new, HVLC surgical hubs in England were initially proposed in orthopaedic surgery as part of a ‘bundle’ of interventions by the ‘Getting it Right First Time’ (GIRFT) programme,^4^ who now provide oversight and an accreditation scheme for new surgical hubs. GIRFT anticipated that by ring-fencing facilities and staff, hubs could reduce variation, save money, boost staff morale, better prevent infections and bring about better clinical outcomes.^5^ With backing from both major UK political parties^6 7^ and the Royal College of Surgeons,^8^ the current surgical hubs programme came with potential access to a £1.5 billion Targeted Investment Fund to expand elective capacity.

Despite no formal requirement to adopt hubs, national rollout has been rapid: 100 NHS trusts now operate a hub, with 26 more planned for 2025.^9^ While a large independent evaluation is underway,^10^ the programme was introduced without prior independent evidence, with stakeholders acknowledging that decisions were not ‘informed by research’.^11^ It therefore echoes other large-scale NHS reconfigurations such as the utilisation of general practitioners in emergency departments, NHS 111, Primary Care Networks and Vanguard new care models.

Research on these initiatives highlights that success or failure depends less on technical design and more on adaptive capacity, relational work, organisational readiness and sustained infrastructure.^12^,^22^ They point particularly to the need for clear policy goals and national backing combined with targeted resourcing,^13 17 22^ organisational readiness and workforce capacity with strong leadership or local championing,^13 14 19 22 23^ and flexibility to tailor central principles to variable local circumstances.^12^,^1422^

This study examines the implementation of surgical hubs in England from the perspective of senior leaders involved in their development and rollout. As key informants, these provide high-level insight into how hubs are both thought of and acted on in policy and practice.^24^ By focusing on system-level decision-making and organisational mechanisms, we extend existing evidence on how large-scale, policy-driven reconfigurations are negotiated and adapted in practice.

### Objectives

To describe the key components of the HVLC surgical hubs programme’s implementation in England from the perspective of key informants in the programme’s development and delivery.

## Methods

This is an interpretive study, conducted as part of the MEASURE (Mixed Methods Evaluation of the high-volume low-complexity surgical hUb pRogrammE) project, funded by the National Institute for Health Research, Health Services and Delivery Research Programme (NIHR153387). Full study details can be found in our mixed methods protocol paper.^10^ Data comprised semistructured interviews with key informants. Ethical approval was obtained from the East Midlands-Nottingham Research Ethics Committee (23/EM/0231). The study followed Standards for Reporting Qualitative Research guidelines for qualitative research.^25^

### Patient and public involvement

As this study engages directly with the development and implementation of surgical hub policy, no patients or members of the public were involved in its development. The broader MEASURE study—and therefore the study aims and objectives built on here—incorporated public and patient involvement.

### Data collection

We conducted 12 interviews with NHS key informants, selected purposefully for their direct involvement in the development and/or implementation of the HVLC surgical hubs programme. Contact with those chiefly responsible for delivery of the hub programme was made when developing the funding application for the MEASURE project. The remaining interviewees were identified via snowball sampling, relying on the contacts of the senior service leaders or members of the MEASURE study steering committee and project advisory group.^26^

Respondents were all NHS employees and included those responsible for national programme delivery (executive and implementation managers), national managers, senior hospital managers, regional NHS managers, and consultants across surgical fields and perioperative care/anaesthetics. Several respondents held or had held executive and directorship positions within the NHS. The level of experience of the sample and their involvement in the surgical hub programme provided information-rich data. Coupled with experienced qualitative researchers and a backdrop of established theory in qualitative health research—with interview guides informed by the Consolidated Framework for Implementation Research (CFIR)^27^—this resulted in a high level of ‘information power’, meaning a sample size of 12 was deemed appropriate.^28^

We used semistructured interviews, conducted online using an interview guide with areas of interest derived from policy-focused literature regarding health, surgery and policy documents relating to surgical hubs (see online supplemental file). The interview guide was flexible to account for the different professions and roles of the key informants in relation to the surgical hubs. Interviews were conducted by PL and AS. These were recorded using the online video-conferencing software’s recording facility, before being manually transcribed by PL.

### Analysis

Analysis was led by PL, with regular discussions held with JA and AS throughout theme development and interpretation. All authors are non-clinical academics. PL is an experienced social scientist and methodologist with no prior experience of surgical research. JA and AS are senior mixed methods researchers with experience of and a particular interest in surgery and qualitative methodology.

Our analysis focused on describing the development and implementation of the HVLC programme from the perspective of those who pioneered and conceptualised the directive nationally. We analysed our data through Braun and Clarke’s thematic analysis approach,^29^ where we adopted an interpretive approach to our analysis that was based on ‘telling the story’ of the surgical hubs programme’s inception and implementation.^30^ We first familiarised ourselves with interview data by repeatedly reading transcripts. We then undertook initial coding in NVivo 12, developing a draft codebook iteratively by moving between the data, existing implementation frameworks (eg, CFIR) and previous literature on health service reconfiguration. Throughout this process, particular attention was paid to understanding how hubs have been so rapidly scaled-up and adopted and to explore the underpinning philosophy and ambitions for the directive. We used flexible coding,^31^ with the codebook (including index codes and analytical codes) refined through regular team discussions, drawing on disciplinary expertise in sociology, psychology and health services research, as well as practical experience of surgical work through consultation with the wider MEASURE study team. Quotes were generally selected based on how well they illustrated broader themes; however, some were more unique, capturing the views of those with more power in the implementation process.^28^

All data are presented anonymously. Throughout the results section, we have highlighted whether participants identified as having predominantly clinical or managerial roles. Further details have been withheld to maintain confidentiality and anonymity of our sample. This is with the exception of one participant who gave explicit consent to be quoted directly and acknowledged that this may make them identifiable.

## Results

Several interrelated themes help explain the widespread diffusion of the HVLC surgical hubs: the timely intervention of high-level clinical leaders in a rapidly changing context; clinically generated data and guidelines developed through ongoing dialogue; an iterative implementation model linking broader strategy with operations at the local level; and an accreditation process that supports implementation, through the sharing and showcasing of best practice and the refining of clinical thresholds.

### Shifting context

A changing context strongly influenced the implementation of the surgical hubs programme. Despite successful surgical hub pilots across various NHS sites prior to the COVID-19 pandemic, there was a lack of interest from the commissioning board, NHS England (NHSE). To respondents, slow and overly bureaucratic processes also hindered innovation. This changed following the COVID-19 outbreak in England and the postponement of elective care. Rapidly increasing waiting lists—and the need to ring-fence non-infected from infected patients—led to interest from NHSE.

The shock provided by the pandemic opened a ‘policy window’ in healthcare systems around the world,^32^ presenting what one respondent described as a ‘real game changer’ for implementation (Clinician, SL0421). By turning the chronic problem of steadily growing waiting lists into an acute crisis with elective surgical care postponed altogether, respondents explained that it ‘released the need to do something differently’ (SL0421). This obligation opened the door for an embryonic surgical hub programme, following the example set by GIRFT:

“(The Regional NHS Director for London) said “we want you to work with us to develop (the Nightingale) and extend the hub sites across London”, so that’s what we did (…) We changed government policy, and the GIRFT programme set the seeds for that” (Clinician*,* SL0525)

To develop capacity to meet this new landscape, targeted funding (through the Targeted Investment Fund) was channelled from other priorities to surgical hubs. With guidance developed by GIRFT—formulated as an attempt to challenge what some saw as a historically disparate, sporadic and unaccountable NHSE funding system—surgical hubs secured around £1 billion of this fund.

Key informants also described how the immediacy of this new context created a more productive mindset; that in this time during the pandemic, “(t)he NHS were able to do some things in two weeks that (…) would typically take them ten years” (Management, SL0625). This involved a different approach to risk: a temporary ‘good enough mentality’, where “not making a decision is worse than making the wrong decision” (SL0625). While this is an extreme position, taken at the height of the pandemic, key informants were keen to insist on the stark difference from what they saw as the usual risk-averse culture:

“…some of the guidelines that we have restrict innovation because we’re worried about risk, and we hide behind risk, and in COVID we were allowed to step out of that” (Clinician, SL0421)

The shifting context therefore brought a clearer demand for change, more funding, and a mindset and approach to risk better suited to innovation.

### Credible clinicians and a clinically produced evidence base

Respondents described the central role of ‘credible clinicians’ (Management*,* SL07) and high-level clinical leaders. This included a founder of the GIRFT programme, which provides some oversight of the surgical hubs, predominantly through the hub accreditation programme. The founder’s drive for standardised, data-led surgical care was well suited to the new surgical landscape following the disruptions of COVID-19 and most key informants mentioned his influence and leadership. This is put down to the GIRFT founder’s ‘clout’ from being President of the British Orthopaedic Association as well as executive roles in the NHS. This influence served as a more facilitating backdrop to discuss data and potential improvements when visiting sites:

“…not only did (they) go there with data, (they) went there with the clout to be able to drive it, because people knew (their) reputation as a clinician, as data-driven (…) So already, you were in a very good frame of mind to show data, sometimes which was great, some which challenged what they were doing, but (they) could have a grown up conversation with those individuals, to talk about how they could improve their services” (SL0525)

A data-led approach, to the key informants, was fundamental to implementation, drawing support from across the clinical community and from policymakers.

This approach involves hubs receiving a ‘data pack’^33^—a dashboard of outcome, productivity and financial metrics for self-assessment against ‘top decile’ performers. These data are described by respondents as ‘crucial’ to convince those reluctant to change their practice:

“…it’s got to be evidence based. So over time when you can show the quality outcomes of working in a different way, that starts to win over the clinicians that might not have believed it in the first place, because if you have worked and you’ve always taken five day length of stay for an arthroplasty and you’ve had a great career and that’s when it’s been suddenly changed to one day or zero day length of stay, it’s a big step change, isn’t it? So you want to see the evidence” (Management*,* SL11)

Respondents were clear that while the evidence base itself is important, as important was its origin in, and development through, clinical practice. For GIRFT-supported surgical hubs, specialty teams are expected to follow the protocols outlined in earlier GIRFT reviews of variation, developed by senior (‘credible’) clinicians, in relation to performance metrics amalgamated from NHS data sources. This type of evidence—developed by senior, and often known, colleagues—is trusted:

Interviewer: “… how have you got that buy-in (from surgeons)?”

SL0525: “The data. The clinical conversation, led by clinicians. That’s what has been missing. They believe in it, they trust it.”

Though led by clinicians, this clinician describes a ‘shoulder-to-shoulder’ approach, with practice being “…clinically led, with data (…) then you use the managers to wrap around to provide you with the tools to do the job well” (SL0525).

Working ‘shoulder-to-shoulder’, the inclusion of those in management, operations and clinicians—referred to as ‘the triumvirate’ by respondents—in the development and implementation of hubs was another important facilitator reported by key informants, enabled by this data-led approach. While a few respondents spoke of concern over safety in hubs at a more general level, reflecting the dominant litigious culture, a larger number of interviewees felt the locus of data offered a way to push back against a cultural aversion to risk. Surgeons spoke of the hubs as a space to ‘prove the concept’, testing and stretching the clinically produced guidelines, with more complex procedures and patients classed as higher risk.

### A responsive mode of implementation with a culture of ‘improvement’

One potential hindrance to implementation, according to service leaders, was the heterogeneity of the surgical hubs:

“…there isn’t a ‘one size fits all’. Big inner-city models are different than rural models, and if you’re a multi-site organisation, you’ve got solutions versus if you’re in a big landlocked city centre site, so there has to be some nuance, some local adaptation” (SL07)

To key informants, GIRFT met this need for local adaptation with ‘deep dive’ visits, adapting broader guidelines (eg, GIRFT procedure protocols) to the local site. Spending “a lot of time out on the floor” connecting “the operational side (…) up to strategy level” (SL0625), this included tailoring protocols to the context of local resources, the existing organisation of services and the needs of the local population. A responsive approach, proactively assessing the needs of local sites, improved engagement with implementation, with respondents contrasting this with what they saw as NHS England’s consistent preference for top-down implementation.

These ‘deep dive’ visits also occurred “in an improvement way, not as an inspection” (Management, SL0118), helping to remove any sense of top-down implementation, as evident in the latitude given to sites in reaching targets for accreditation:

“We’re not absolutely definitive about everything. There are things like theatre utilisation, for example, where we say you must be running your theatres 48 weeks a year, six days a week, and three sessions a day, but right at the beginning we said that a lot of hubs won’t be able to be part of this process, so what we’ve said is that you must be working towards that, and be able to demonstrate that you’re working towards it. So that doesn’t exclude them, but allows hubs to have a certain amount of autonomy (…) I think sites are pleasantly surprised that this isn’t something about beating them up” (Management*,* SL0224)

Other NHS England modes of oversight were seen as taking an authoritative performance management approach, pulling performance into step with exemplar sites by automatically penalising variation. GIRFT had exemplar-based targets, but recommendations were based on an offer of help. Where “other parts of the NHS will just shout at you louder” (SL0625), respondents suggested that this approach meant site teams were more likely to engage with the process.

One member of GIRFT’s implementation team described the importance of balance between broader guidelines and local adaptation, referring directly to ‘top down’ and ‘bottom up’ approaches:

“I would slightly facetiously call it a ‘pincer movement’. So you have to have both. If we just went round the country saying “here, you’re having a hub”, just absolutely no chance (…) …what we’re doing is providing a framework that people can apply locally, you know – we’re not telling them exactly what a hub is. Part of the journey with the systems has been going, OK what’s your hub plan? (…) So actually, it feels like bottom up because we’re talking to people, understanding what they need, and challenging it – not just taking it at face value, we’re challenging a lot of stuff” (SL0625)

This speaks to an apparent iterative approach to implementation, with targets and challenges a part of the clinical conversation with staff during ‘deep dive’ visits. An example of this is provided by a member of the GIRFT implementation team attempting to ‘sell’ protocols for higher complexity patients around the country, in a culture that often leans toward risk aversion:

“…just take ASA-3 and −4 patients: that’s taken ‘whites-of-the-eye’ conversations. We have to drive to the hospital, sit down with the anaesthetist, have to talk them through… (…) Like, you can’t shortcut some of those conversations and therefore, you know, we’ve just literally had to tour the country (…) one anaesthetist at a time, as it gets taken up. But of course what you get, once you start to get the group, and then it becomes the laggards and then, you know, it’s kind of moving through that change curve, and the other thing is using those early guys as the proponents, they’re the cheerleaders, so it’s not coming from me, it’s coming from the guy that was really sceptical about it, and now he’s telling people he wouldn’t go back” (SL0625)

### Peer-to-peer, localised accreditation

The accreditation programme was set up to maintain a sense of control and financial accountability as the hub programme expanded and targeted funding was awarded. It assesses hubs across five criteria: the patient pathway; staff and training; clinical governance and outcomes; facilities and ring-fencing; and utilisation and productivity.^5^ While each requires a lot of work by the hubs, it was apparent that each criterion retains enough breadth for successful accreditation across heterogeneous sites.

To one surgeon, accreditation fills a broader staff satisfaction gap, providing an opportunity to ‘capture’ work already being done behind the scenes: “it mandates that someone sits and pulls together thought processes and that in itself is really helpful” (SL0421). For patient confidence and trust, accreditation was seen to provide ‘kudos’ (Management*,* SL08) and legitimacy, and that patients would be offered a ‘premium product’ (Management, SL0224). For those areas of the country with larger and still increasing waiting lists, a premium product was considered important to tempt patients to travel to a productive surgical hub:

“…if you’re an elective hub and you’re churning through your activity and you can offer mutual aid to other areas, you need to be able to influence those patients’ decision making that they’re going to somewhere special” (SL0421)

Beyond these benefits, the actual process of accreditation was perhaps the most important facilitator for engagement with surgical hubs and implementation. GIRFT included staff—operational, managerial and clinical—from hub sites undergoing, or already having undergone accreditation, into the team for ‘deep dive’ accreditation visits. Doing so, they indirectly built a network of engaged and supportive surgical hub peers. This was described as ‘a touch of genius’ (SL0625), leading to a ‘groundswell’ of engagement (SL0118). Largely for this reason, every key informant interviewed spoke highly of the accreditation programme, with most mentioning the ever-growing list of clinicians and managers wanting to join visits. To one consultant surgeon, the processes served to share best practice and highlight responses to collective problems:

“You really get to learn best practice and share best practice, and see how other people have overcome certain challenges, because most of the challenges in the health service are similar, (though) they may be slightly different due to regional or geographical variation” (Clinician, SL0329)

Adding peers to the process of implementation—through the multiplication of potential feedback loops—allows the sharing of best practice, providing opportunities to distribute ‘tacit knowledge’ and experiential learning, while also building a network of interested contacts, more likely to buy-in to the surgical hubs programme.

### Discussion

This paper explores the implementation of the HVLC surgical hubs programme in England. COVID-19 served as the backcloth to several critical factors: the role of credible clinical staff; the embedded use of clinically produced data sets; improvement-focused oversight, responsive to local needs and capacities; and a supportive surgical hub network fostered through accreditation. While these are novel insights in the study of the surgical hub programme, they speak to years of research elsewhere. Highlighting this research and examples from this study, table 1 therefore reiterates a series of recommendations for the implementation of future health service reconfigurations.

**Table 1:** Eight recommendations for future health policy development and implementation

Domain	#	Recommendation	Example
Pre-existing policy structures	1	Leverage existing initiatives and identify ‘windows of opportunity’ to accelerate adoption^16 32^. Crisis conditions can remove bureaucratic barriers and create urgency^36^.	The surgical hubs programme built on GIRFT’s earlier work to reduce variation. COVID-19 created a policy window that enabled rapid acceptance and engagement.
Credibility and resonance	2	Secure involvement of credible individuals trusted by those whose practice will change^23 40^. Leadership ‘clout’ and peer legitimacy are critical for buy-in.	The reputation of respected clinicians and clinical leaders was pivotal in engaging surgical teams.
	3	Align policy control with clinicians’ existing practices and values to sustain engagement^45 54^. Evidence developed within a ‘community of practice’ resonates more than centrally imposed guidelines.	Practice-based evidence from GIRFT reviews secured engagement, even among initially reluctant surgeons.
Oversight	4	Adopt improvement-focused oversight rather than punitive performance management, fostering shared ownership and incremental progress^46 51^.	Accreditation framed as supportive dialogue allowed sites to meet targets while feeling empowered as equal partners.
	5	Ensure oversight is responsive to local context, including population needs, facilities and workforce^15 48^. Flexibility encourages engagement and innovation.	GIRFT adapted protocols during ‘deep dive’ visits, tailoring guidance to local realities.
Dialogue and network-building	6	Embed structured dialogue and feedback loops throughout implementation^52 53^. Peer exchange accelerates learning and diffusion of innovation^54^.	Accreditation visits included staff from other hubs, creating a learning environment and opportunities for shared innovation.
	7	Showcase existing work and share best practice to build legitimacy and morale^18^. Recognising local efforts fosters ownership and engagement.	GIRFT emphasised highlighting staff achievements and aligning guidance with existing initiatives.
	8	Develop infrastructure to sustain peer networks beyond initial implementation^52 54^. Networks enable ongoing problem-solving and reduce reliance on central oversight.	Accreditation’s snowball approach fostered a growing peer network, enabling hubs to share advice and innovation beyond GIRFT oversight.

GIRFT, Getting it Right First Time.

The COVID-19 pandemic created a ‘window of opportunity’ in healthcare, creating both the rationale and the conditions for uptake of surgical hubs. On the one hand—and similar to other service reconfigurations, such as general practice video consultation,^34^ Primary Care Networks,^17^ NHS 111^20^ and NHS 111 online^21^—it created a specific need: in this case, the demand for dedicated ‘green sites’, often the precursor to surgical hubs. Following COVID-19, staff therefore had familiarity with the concept of ring-fencing^35^ and greater organisational readiness. On the other hand, the crisis conditions of the pandemic removed bureaucracy, necessitating innovation and a more open, less risk averse approach. Although these conditions cannot be replicated, future policy could simulate similar ‘system resets’, providing time and space to approach change^16^—for example, through coordinated pressure for change^36^ or mechanisms to prompt thinking beyond organisational boundaries.^37^

The surgical hub programme is underpinned by the GIRFT programme, which draws on clinically generated data, including Hospital Episode Statistics and reviews of practice variation. Developed by credible, often known, clinicians, this evidence is trusted by clinical, managerial and operational staff, and serves as a focus for evaluation and dialogue with local sites. Consistent with research on evidence use in surgical care, trust in this peer-produced evidence—produced by a ‘community of practice’ rooted in local clinical networks and hierarchies^38 39^—resonates more than centrally issued guidelines.^3840^,^42^ While research shows that surgeons can be resistant to standardisation,^40 43 44^ this standardised evidence, accumulated through the work of colleagues, to service leaders seems to be pivotal to the successful rollout of surgical hubs. Questions remain over the longer-term effectiveness of this form of evidence over more centralised guidelines,^45^ and future research should explore the nuances of how these data are used by different clinicians.^43^

A recurrent theme among implementation studies is the tension between top-down or centralised vs bottom-up or decentralised imperatives and approaches to implementation.^46^ Numerous studies cite the need for greater attention to local context in the process of broader service reconfiguration: whether paying more attention to local populations,^19 47^ local representatives or commissioners,^35 48^ local political machinations^15^ or service arrangements.^13 20 49 50^ Service leaders were cognisant of this challenge, foregrounding what might be considered a hybrid approach (one service leader’s ‘pincer movement’). This hybrid model employs authoritative, target-based mechanisms, but these are tempered—sometimes obscured—by persuasive strategies that foster feelings of ownership and consensus within hubs. Its collaborative structure echoes partnership-based models, using feedback, benchmarking and shared learning to drive improvement.^18^ Yet, as Martin and Dixon-Woods^51^ caution, such models often depend heavily on the enthusiasm, judgement and persistence of individual leaders, meaning their impact may hinge on ongoing leadership ‘clout.’

Numerous persuasive strategies, such as through the accreditation scheme, were outlined by service leaders. These included continuous engagement with local sites, accentuated as important elsewhere in healthcare reconfiguration research^52 53^; an emphasis on improvement rather than penalisation for missed targets, similar to what other reconfiguration research has described as pro-active support^36^ or a permissive policy approach^12^; and the ongoing development of a peer-to-peer network, providing channels for legitimacy, credibility and the diffusion of new practices.^16 23 54^

A key strength of this study lies in the richness of its data. As part of the MEASURE project, we were able to engage key informants directly involved in the development and implementation of England’s elective recovery policy. Although the sample size was necessarily limited—reflecting the small number of individuals involved in implementing surgical hubs—it provided access to first-hand experience and detailed insight into their national rollout. The perspectives of key informants depicted an overwhelmingly positive view of the surgical hub programme and its implementation. Despite efforts to identify dissenting voices, these proved difficult to locate through snowball sampling, which may be considered a limitation of the study. This study nevertheless represents the initial phase of the broader mixed-methods MEASURE project.^10^ The subsequent qualitative phase will comprise in-depth case studies of surgical hub sites to examine their functioning and implementation in practice, situating these findings alongside the perspectives of other stakeholders, including surgical hub staff and patients.

## Conclusion

The implementation of ‘high volume, low complexity’ surgical hubs in England illustrates how large-scale reconfiguration can be accelerated under crisis conditions and sustained through a combination of clinical credibility, trusted data and improvement-focused oversight. Our findings highlight the value of hybrid governance approaches that combine national direction with local adaptability, supported by peer networks and accreditation mechanisms. These features align with broader evidence on health system change, emphasising organisational readiness, relational work and the creation of supportive infrastructures over purely technical design.

While the pandemic created a unique ‘window of opportunity’, future policy could replicate some enabling conditions—such as protected time for planning, permissive regulatory environments and mechanisms to foster cross-organisational collaboration—without relying on crisis. Further research should examine how surgical hubs function in practice, how the model is experienced and perceived by those employed by the NHS, their impact on equity and outcomes, and the sustainability of the model beyond initial investment and leadership enthusiasm.

## Supplementary material

10.1136/bmjopen-2026-119396 online supplemental file 1

## Data Availability

Data are available upon reasonable request.
