# Supplementary material for: Understanding implementation of the ‘high volume, low complexity’ surgical hubs programme in England: a qualitative study
Source: BMJ Open. 2026 Jul 17;16(7):e119396. doi: 10.1136/bmjopen-2026-119396 (PMC13384122; doi:10.1136/bmjopen-2026-119396)
Supplement: online supplemental file 1 [file bmjopen-16-7-s001.docx]

**Understanding implementation of the ‘high volume, low complexity’ surgical hubs programme in England: a qualitative study**

**Supplementary File**

**Service Leader Interview Guide**

Core project questions as well as prompts. As interviews are semi-structured, other questions may be asked.

**About them**

- Can you start by describing your role - what it is you are responsible for?

**Policy development and delivery**

- Why are surgical hubs needed?
- We're interested in the *story* of the elective hubs policy, so can you tell us a bit about its development?
- How has the policy developed over time?
- Has there been any changes in remit?

**Individuals**

- Who is driving/leading the policy?
  - Policy level? At sites?
- Who is delivering the policy on the ground?
  - Is anyone else involved?

**Evidence**

- What evidence is being used to inform the implementation of surgical hubs?
- What informed it to begin with? Did it evolve out of something else?
- How are decisions around the surgical hubs and their implementation being made?

**Comparable policy implementation**

- Are there any comparable programmes?
- Would you describe it as closer to bottom up or top down implementation?

**Implementation**

- Are there differences in how the policy is implemented across different sites?
- Any particular facilitators for implementation? *What has helped?*
- Any barriers to implementation? *What are the biggest difficulties?*
- Are there any transferable lessons to other implementation efforts?

**Patients**

- Have you had any patient feedback on surgical hubs?
- In what way are patients considered in the planning of surgical hubs?

**Implementation success**

- Is there any reluctance or resistance? Over anything particular?

**Funding**

- How is the surgical hubs programme funded?
- How does the funding (funding channels) work?
- Any additional funding?
- Are there competing initiatives for the funding?
- Is there accountability for the funding?

**Independent sector**

- How much is the independent sector involved in the hubs?

**Accreditation**

- Can you tell me about the accreditation programme?
- How did the idea of accreditation come about?
- How has accreditation been received?

**Impact**

- How would you describe the impacts of the hubs?
- How are impacts evaluated?
- What KPIs or data do you collect?
- How are these distributed back to stakeholders?
- Any Spillover effects?
