# Supplementary material for: Understanding implementation of the ‘high volume, low complexity’ surgical hubs programme in England: a qualitative study
Source: BMJ Open. 2026 Jul 17;16(7):e119396. doi: 10.1136/bmjopen-2026-119396 (PMC13384122; doi:10.1136/bmjopen-2026-119396)
Supplement: Supplementary file 2 [file bmjopen-2026-119396.reviewer_comments.pdf]

PEER REVIEW HISTORY

BMJ Open publishes all reviews undertaken for accepted manuscripts. Reviewers are asked to complete a checklist review form and are provided with free text boxes to elaborate on their assessment. These free text comments are reproduced below.

ARTICLE DETAILS

Title (Provisional)

Understanding implementation of the 'high volume, low complexity' surgical hubs programme in England: a qualitative study

Authors

Lampard, Pete; Scantlebury, Arabella; Sivey, Peter; Adamson, Joy

---

VERSION 1 - REVIEW

**Reviewer**	**1**
**Name**	**Sanjay Beesoon**
**Affiliation**	**Alberta Health Services**
**Date**	**08-Sep-2025**
**COI**	**None**

The whole manuscript needs to be re-written in a logical and scientific manner.

General Comments: The implementation of HVLC hubs in England a bold and innovative idea that the NHS system implemented despite the lack of evidence showing its effectiveness to increase volumes of surgeries and thus decrease wait times. It is obvious that the implementation was not an easy journey and there was significant learning by navigating both top-down and bottom-up strategies.

The authors gathered a lot of rich data, but unfortunately the manuscript has not been presented in a logical and scientific manner. After a couple of rounds of reading the material, it looks more like an informational paper rather than a scientific manuscript that describes a process that other health systems can adapt and replicate to improve the delivery of surgical care in publicly funded healthcare systems. Extensive direct quotes from the interviewees throughout the paper, without a robust qualitative analysis of the emerging themes.

I suggest re-writing the paper with the following:

1. **Background** – Why was it important to introduce HVLC – High demand-low supply getting worse with COVID. Please give numbers of (1) wait times, (2) number of surgeons – struggles with recruit and retaining (3)

inflating costs of providing surgical care. The rational for introducing HVLC

2. **Objective(s)** : Why did the team decide to interview the senior leaders? Was it to learn about the barriers and opportunities of introducing HVLC? Was the intent to learn about mistakes made and to course-correct? Was it a process evaluation?
3. **Methodology**: How were participants identified? Was ethics approval sought? How was the questionnaire developed? How was the interviews conducted – were they recorded? Who transcribed these? Was special software used for transcription? Was software used to analyze the data? What was the process to develop themes in the context of CFIR The updated Consolidated Framework for Implementation Research based on user feedback | Implementation Science | Full Text
4. **Results**: What were the findings – (1) The common themes that emerged based on the analysis (2) Some main quotes from the interviewees (3) Major successes
(4) Major barriers
5. **Discussion**: interpretation of the findings and learnings (here the concept of Learning Health System Actioning the Learning Health System: An applied framework for integrating research into health systems - ScienceDirect can be

   applied. Discuss whether it is always necessary to have peer reviewed research before implementing innovations in health systems. Describe how crisis situations, like COVID, force health leaders to innovate. What was the the Return on Investment for HVLC
6. **Limitations of the study**: Sample Size – Interviews limited to senior leadership – No patient voice
7. **Conclusion and recommendations**: from the perspective of health leaders, was the implementation of HVLC hubs a success – what are the recommendations of the authors for future versions of HVLC. How can other medical specialties learn and adopt from HVLC hubs.

My Recommendation:

I recommend the paper be re-written (major revisions) and re-submitted to BMJ Open

---

**Reviewer**	**2**
**Name**	**Laurent Meriade**
**Affiliation**	**University of Clermont Auvergne**
**Date**	**08-Sep-2025**
**COI**	**None**

The article submitted for my review is entitled 'Understanding implementation of the 'high volume, low complexity' surgical hubs programme in England: a qualitative study'.

In terms of form, the article complies with the presentation standards for a scientific article. The arguments are clearly presented and easy to understand.
In terms of content, in the introduction, the authors state that in this article they explore the introduction of the HVLC (high volume, low complexity) surgical hubs programme in England and identify the key elements of the implementation of this policy that could shed light on our understanding of the 'successful' development and implementation of a health policy. The ambition is noble, but in my opinion it suffers from numerous shortcomings, particularly conceptual but also methodological, which I will outline below:
1° The ultimate objective of the article is to present at the end of the introduction: 'To describe the key components of the HVLC surgical hubs programme's implementation in England from the perspective of key informants in the programme's development and
delivery.' In practical terms, this objective is particularly interesting and stimulating. It should make it possible to present the specific features of the implementation of an HVLC centre. On the other hand, from a conceptual point of view, the research objective is poorly argued. What is the literature on which this article is based? What will this article contribute? What research gap will it fill? I believe it is essential, in the introduction to the article, to indicate the current state of the literature on the implementation of health or surgical systems and to mention what gap this article is likely to fill. In my opinion, without this information, the article loses its ability to convince both the evaluators and, above all, the readers of this work. It is imperative in the introduction to review the literature on the implementation of health or surgical facilities. Numerous studies have already examined and analysed the implementation of surgical devices in depth, for example: Maru et al. 2011; Alidina et al., 2018; Klaic et al., 2022; Ikpot et al., 2025; Lambert-Kerzner et al., 2025).

2° The methodology used is rich and relatively well detailed. However, it seems to me that a more precise description of the people interviewed (exact roles, experience, duration of the interview, etc.) would give a clearer idea of the consistency between the research objective and the research method used. The authors simply state that twelve interviews were conducted with key informants from the NHS directly involved in the development and/or implementation of the HVLC programme, but their exact roles are not known. They add that the other interviewees included those responsible for implementing the national programme (managers and implementation managers), national directors, senior hospital managers, regional NHS directors and consultants in the fields of surgery and perioperative/anaesthetic care. The number of these other interviewees is unknown, as are the characteristics of the institutions in which they work. It is therefore difficult to understand how snowball sampling was carried out. This should also be explained.

3° The data was coded using Nvivo, but it is difficult to understand how this coding was carried out. Was it open coding? Axial coding? Selective coding? It would be useful to specify how this coding was carried out or even to present the coding grid, otherwise it is difficult to assess the consistency of the research approach.

4° The results are interesting. They trace the main verbatim quotes through four main themes: Change of context with COVID-19, Credible clinicians and a clinically produced evidence base, Responsive mode of implementation with a culture of "improvement", Peer-to-peer, localised accreditation. The structure of these results seems convincing to me. However, it is difficult to understand how this structure and the verbatim quotes emerged. Not knowing whether an interview grid was constructed for this study, it is difficult to be completely convinced by these results. Can the authors provide their interview grid and explain how the verbatim quotes were selected?

5° The discussion focuses on the presentation of eight recommendations for the future development and implementation of health policies. These eight recommendations are interesting but, in my opinion, they do not relate to health policies. They are limited to the implementation of surgical centres or hubs. This description of the eight recommendations needs to be changed. On the other hand, even if these recommendations are very much rooted in practice, it is difficult to understand how they confirm, refute or enrich previous work on the implementation of surgical facilities. In line with my first comment, it is imperative to discuss these recommendations alongside those formulated in previous work. The authors cite a few articles in this 'discussion' section, but their results were not presented in the introduction, and it is difficult to understand how the article enriches or complements this work. In my opinion, if the following two tasks are not carried out (presentation of a research gap based on an analysis of the

existing literature and discussion of the results with this literature), this article cannot be published.

6° As is often the case, the conclusion of the article does not revisit the theoretical contributions of the article but merely describes the programme studied once again. Once again, I believe that the theoretical and, to a lesser extent, practical contributions of the article are unclear. They should be described and justified

In general, the authors state at the end of their article that 'A key strength of this study was its information rich data:'. I agree with this statement, however, it seems to me that the weaknesses I mentioned above (theoretical and methodological) must be addressed before considering the publication of this work.

VERSION 1 - AUTHOR RESPONSE

Reviewer 1.

I. **Reviewer 1:** Very significant improvement has been made to the manuscript since my first review. The substance of the manuscript as well as the presentation and inference meet the criteria for publication. I am satisfied that the findings of this research have a direct relevance to public policy regarding surgical wait lists.
Response: We thank the reviewer for their comments for helping us to make significant improvements to the manuscript.

Reviewer 2.

II. **Reviewer 2:** The ultimate objective of the article is present at the end of the introduction: 'To describe the key components of the HVLC surgical hubs programme's implementation in England from the perspective of key informants in the programme's development and delivery.' In practical terms, this objective is particularly interesting and stimulating. It should make it possible to present the specific features of the implementation of an HVLC centre. On the other hand, from a conceptual point of view, the research objective is poorly argued. What is the literature on which this article is based? What will this article contribute? What research gap will it fill? I believe it is essential, in the introduction to the article, to indicate the current state of the literature on the implementation of health or surgical systems and to mention what gap this article is likely to fill. In my opinion, without this information, the article loses its ability to convince both the evaluators and, above all, the readers of this work. It is imperative in the introduction to review the literature on the implementation of health or surgical facilities. Numerous studies have already examined and analysed the implementation of surgical devices in depth, for example: Maru et al. 2011; Alidina et al., 2018; Klaic et al., 2022; Ikpot et al., 2025; Lambert-Kerzner et al., 2025).
Response: We are grateful to the reviewer for their comments around the conceptual and literature contribution as it has enabled us to focus the arguments throughout the paper. While the literature suggested by the review is interesting, we have located the field of literature within health service reconfiguration research (rather than research around the implementation of surgical devices referred to by the reviewer). We feel this has allowed us to better argue our research objectives.
We have added literature on large scale health service reconfigurations to the introduction to highlight the literature base the study adds to as well as what research gaps it fills, with this research around a large scale surgical transformation in England [66-74 or 1 below].
We have also drawn on this literature again within the discussion to contextualise our findings [see Box 1; lines 347-350 or 2 below; 362-370 or 3 below; 371-384 or 4 below; 386-391 or 5 below]. (This also relates to points made below under VII).

III. **Reviewer 2:** The methodology used is rich and relatively well detailed. However, it seems to me that a more precise description of the people interviewed (exact roles, experience, duration of the interview, etc.) would give a clearer idea of the consistency between the research objective and the research method used. The authors simply state that twelve interviews were conducted with key informants from the NHS directly involved in the

development and/or implementation of the HVLC programme, but their exact roles are not known.
Response: Regarding description of the people interviewed, the relatively small number of individuals involved in the development and implementation of the surgical hubs programme nationally has prevented us from providing further information (as per the editor's suggestion in the last round of feedback: "Please consider anonymising the quotes, by removing the role of the participant") so as to maintain anonymity and confidentiality of our participants. We have provided further clarification within our methods section [lines 146-150 or 6 below] as to why we were unable to provide further information regarding participants roles.

IV. **Reviewer 2:** They add that the other interviewees included those responsible for implementing the national programme (managers and implementation managers), national directors, senior hospital managers, regional NHS directors and consultants in the fields of surgery and perioperative/ anaesthetic care. The number of these other interviewees is unknown, as are the characteristics of the institutions in which they work. It is therefore difficult to understand how snowball sampling was carried out. This should also be explained.
Response: For further context and clarity, additional information about our sampling method has been added to the methods section [see analysis – lines 99-105 or 7 below].

V. **Reviewer 2:** The data was coded using Nvivo, but it is difficult to understand how this coding was carried out. Was it open coding? Axial coding? Selective coding? It would be useful to specify how this coding was carried out or even to present the coding grid, otherwise it is difficult to assess the consistency of the research approach.
Response: We have provided more information about the coding process within the methods section. Here we outline flexible coding via index and analytical codes, as well as how this was utilised within the research team [lines 133-143 or 8 below].

VI. **Reviewer 2:** The results are interesting. They trace the main verbatim quotes through four main themes: Change of context with COVID-19, Credible clinicians and a clinically produced evidence base, Responsive mode of implementation with a culture of "improvement", Peer-to-peer, localised accreditation. The structure of these results seems convincing to me. However, it is difficult to understand how this structure and the verbatim quotes emerged. Not knowing whether an interview grid was constructed for this study, it is difficult to be completely convinced by these results. Can the authors provide their interview grid and explain how the verbatim quotes were selected?
Response: We have provided the interview guide within the supplementary material with a link to (and discussion of) this in the methods section [line 118-120 or 9 below]. We have added to the methods section information about how quotes were selected, outlining the concept of 'information power' [lines 143-145 or 10 below].

VII. **Reviewer 2:** The discussion focuses on the presentation of eight recommendations for the future development and implementation of health policies. These eight recommendations are interesting but, in my opinion, they do not relate to health policies. They are limited to the implementation of surgical centres or hubs. This description of the eight recommendations needs to be changed. On the other hand, even if these recommendations are very much rooted in practice, it is difficult to understand how they confirm, refute or enrich previous work on the implementation of surgical facilities. In line with my first comment, it is imperative to discuss these recommendations alongside those formulated in previous work. The authors cite a few articles in this 'discussion' section, but their results were not presented in the introduction, and it is difficult to understand how the article enriches or complements this work. In my opinion, if the following two tasks are not carried out (presentation of a research gap based on an analysis of the existing literature and discussion of the results with this literature), this article cannot be published.
Response: We have updated and redescribed the recommendations to align them with previous literature on healthcare [see updated Box 1] as above (item **II**), making them more relevant to health policy implementation more broadly. We have also referred back to the literature base more substantially within the discussion [lines 347-350 or 2 below; 362-370 or 3 below; 371-384 or 4 below; 386-391 or 5 below]

Again, we are very grateful to the reviewer for this recommendation as it has helped us to better contextualise our findings with this literature base.

VIII. **Reviewer 2:** As is often the case, the conclusion of the article does not revisit the theoretical contributions of the article but merely describes the programme studied once again. Once again, I believe that the theoretical and, to a lesser extent, practical contributions of the article are unclear. They should be described and justified.
Response: We thank the reviewer for this helpful comment and have revised the conclusion to include recommendations for future policy implementation, as well as theoretical/practical contributions [lines 406-420 or 11 below].

Evidence

1. "It therefore echoes other large-scale NHS reconfigurations such as the utilisation of general practitioners in emergency departments, NHS 111, Primary Care Networks, and Vanguard new care models.
   Research on these initiatives highlights that success or failure depends less on technical design and more on adaptive capacity, relational work, organisational readiness, and sustained infrastructure [12-22]. They point particularly to the need for clear policy goals and national backing combined with targeted resourcing [13, 17, 22], organisational readiness and workforce capacity with strong leadership or local championing [13, 14, 19, 22, 23], and flexibility to tailor central principles to variable local circumstances [12-14, 22]."
2. The COVID-19 pandemic created a 'window of opportunity' in healthcare, creating both the rationale and the conditions for uptake of surgical hubs. On the one hand – and similar to other service reconfigurations, such as general practice video consultation [34], Primary Care Networks [17], NHS 111 [20], and NHS 111 online [21] – it created a specific need: in this case the demand for dedicated 'green sites', often the precursor to surgical hubs.
3. Consistent with research on evidence use in surgical care, trust in this peer-produced evidence – produced by a 'community of practice' rooted in local clinical networks and hierarchies [38, 39] – resonates more than centrally issued guidelines [38, 40-42]. While research shows that surgeons can be resistant to standardisation [41, 43, 44], this standardised evidence, accumulated through the work of colleagues, to service leaders seems to be pivotal to the successful rollout of surgical hubs. Questions remain over the longer term effectiveness of this form of evidence over more centralised guidelines [45], and future research should explore the nuances of how this data is used by different clinicians [43].
4. A recurrent theme among implementation studies is the tension between top-down or centralised versus bottom-up or decentralised imperatives and approaches to implementation [46]. Numerous studies cite the need for greater attention to local context in the process of broader service reconfiguration: whether paying more attention to local populations [19, 47], local representatives or commissioners [35, 48], local political machinations [15], or service arrangements [13, 20, 49, 50]. Service leaders were cognisant of this challenge, foregrounding what might be considered a hybrid approach (one service leader's "pincer movement"). This hybrid model employs authoritative, target-based mechanisms, but these are tempered—sometimes obscured—by persuasive strategies that foster feelings of ownership and consensus within hubs. Its collaborative structure echoes partnership-based models, using feedback, benchmarking, and shared learning to drive improvement [18]. Yet, as Martin and Dixon-Woods [51] caution, such models often depend heavily on the enthusiasm, judgement, and persistence of individual leaders, meaning their impact may hinge on ongoing leadership "clout."
5. These included continuous engagement with local sites, accentuated as important elsewhere in healthcare reconfiguration research [52, 53]; an emphasis on improvement rather than penalisation for missed targets, similar to what other reconfiguration research has described as pro-active support [36] or a permissive policy approach [12]; and the ongoing development of a peer-to-peer network, providing channels for legitimacy, credibility, and the diffusion of new practices [16, 23, 54].
6. All data are presented anonymously. **Throughout the results section we have highlighted whether participants identified as having predominantly clinical or managerial roles. Further details have been withheld to maintain confidentiality and anonymity of our sample.** This is with the exception of one participant who gave explicit consent to be quoted directly and acknowledged that this may make them identifiable.

7. We conducted twelve interviews with NHS key informants, **selected purposefully for their direct involvement** in the development and/or implementation of the HVLC surgical hubs programme. Contact with those chiefly responsible for delivery of the hub programme was made when developing the funding application for the MEASURE project. The remaining interviewees were identified via snowball sampling, **relying on the contacts of the senior service leaders or members of the MEASURE study steering committee and project advisory group** [26].
8. We first familiarised ourselves with interview data by repeatedly reading transcripts. We then undertook initial coding in NVivo 12, developing a draft codebook iteratively by moving between the data, existing implementation frameworks (e.g., CFIR), and previous literature on health service reconfiguration. Throughout this process particular attention was paid to understanding how hubs have been so rapidly scaled-up and adopted and to explore the underpinning philosophy and ambitions for the directive. We used flexible coding [31], with the codebook (including index codes and analytical codes) refined through regular team discussions, drawing on disciplinary expertise in sociology, psychology, and health services research, as well as practical experience of surgical work through consultation with the wider MEASURE study team.
9. The interview guide was flexible to account for the different professions and roles of the key informants in relation to the surgical hubs. Interviews were conducted by PL and AS.
10. Quotes were generally selected based on how well they illustrated broader themes; however, some were more unique, capturing the views of those with more power in the implementation process [28].
11. The implementation of 'high volume, low complexity' surgical hubs in England illustrates how large-scale reconfiguration can be accelerated under crisis conditions and sustained through a combination of clinical credibility, trusted data, and improvement-focused oversight. Our findings highlight the value of hybrid governance approaches that combine national direction with local adaptability, supported by peer networks and accreditation mechanisms. These features align with broader evidence on health system change, emphasising organisational readiness, relational work, and the creation of supportive infrastructures over purely technical design.
    While the pandemic created a unique 'window of opportunity', future policy could replicate some enabling conditions – such as protected time for planning, permissive regulatory environments, and mechanisms to foster cross-organisational collaboration – without relying on crisis. Further research should examine how these hubs function in practice, how the model is experienced and perceived by those employed by the NHS, and their impact on equity and outcomes, and the sustainability of the model beyond initial investment and leadership enthusiasm.

VERSION 2 - REVIEW

Reviewer 3

Name Zhang, Yongkang

Affiliation Cornell University Joan and Sanford I Weill Medical College, Population Health Sciences

Date 23-May-2026

COI None

Thank you for the opportunity to review this revised manuscript. The authors were very responsive to the reviewers' comments and have made significant revisions. I think the manuscript has been largely improved. I do not have any other comments.

VERSION 1 - AUTHOR RESPONSE

Many thanks - I am resubmitting to answer the questions that were not required at the initial submission.
